# Exploring the Association Linking Head Position and Sleep Architecture to Motor Impairment in Parkinson’s Disease: An Exploratory Study

**DOI:** 10.3390/jpm13111591

**Published:** 2023-11-10

**Authors:** Oriella Gnarra, Carmen Calvello, Tommaso Schirinzi, Francesca Beozzo, Claudia De Masi, Matteo Spanetta, Mariana Fernandes, Piergiorgio Grillo, Rocco Cerroni, Mariangela Pierantozzi, Claudio L. A. Bassetti, Nicola Biagio Mercuri, Alessandro Stefani, Claudio Liguori

**Affiliations:** 1 Sleep-Wake-Epilepsy Center, Department of Neurology, University Hospital of Bern, 3010 Bern, Switzerland; oriella.gnarra@hest.ethz.ch (O.G.); claudio.bassetti@insel.ch (C.L.A.B.); 2 Sensory-Motor Systems Lab, Institute of Robotics and Intelligent Systems, Department of Health Sciences and Technology, ETH Zurich, 8092 Zurich, Switzerland; 3 Department of Systems Medicine, University of Rome “Tor Vergata”, 00133 Rome, Italy; carmen.calvello91@gmail.com (C.C.); t.schirinzi@yahoo.com (T.S.); francesca.beozzo@students.uniroma2.eu (F.B.); pierantozzim@gmail.com (M.P.); mercurin@med.uniroma2.it (N.B.M.); stefani@uniroma2.it (A.S.); 4 Parkinson’s Disease Unit, University Hospital of Rome “Tor Vergata”, 00133 Rome, Italy; claudia.dem7@gmail.com (C.D.M.); piergiorgiogrillo90@gmail.com (P.G.); rocco.cerroni@gmail.com (R.C.); 5 Santa Maria della Stella Hospital, 05018 Orvieto, Italy; spanettamatteo95@gmail.com; 6 Neurology Unit, University Hospital of Rome “Tor Vergata”, 00133 Rome, Italy

**Keywords:** sleep profiler, PD, glymphatic system, head position, slow wave activity

## Abstract

Patients with Parkinson’s disease (PD) tend to sleep more frequently in the supine position and less often change head and body position during sleep. Besides sleep quality and continuity, head and body positions are crucial for glymphatic system (GS) activity. This pilot study evaluated sleep architecture and head position during each sleep stage in idiopathic PD patients without cognitive impairment, correlating sleep data to patients’ motor and non-motor symptoms (NMS). All patients underwent the multi-night recordings, which were acquired using the Sleep Profiler headband. Sleep parameters, sleep time in each head position, and percentage of slow wave activity (SWA) in sleep, stage 3 of non-REM sleep (N3), and REM sleep in the supine position were extracted. Lastly, correlations with motor impairment and NMS were performed. Twenty PD patients (65.7 ± 8.6 y.o, ten women) were included. Sleep architecture did not change across the different nights of recording and showed the prevalence of sleep performed in the supine position. In addition, SWA and N3 were more frequently in the supine head position, and N3 in the supine decubitus correlated with REM sleep performed in the same position; this latter correlated with the disease duration (correlation coefficient = 0.48, *p*-value = 0.03) and motor impairment (correlation coefficient = 0.53, *p*-value = 0.02). These preliminary results demonstrated the importance of monitoring sleep in PD patients, supporting the need for preventive strategies in clinical practice for maintaining the lateral head position during the crucial sleep stages (SWA, N3, REM), essential for permitting the GS function and activity and ensuring brain health.

## 1. Introduction

Sleep disturbances represent a common non-motor manifestation of Parkinson’s disease (PD) since they are reported by 75–80% of PD patients [1,2,3]. REM sleep behavior disorder (RBD), restless legs syndrome, insomnia, nocturia, sleep-disordered breathing, and excessive daytime sleepiness (EDS) are the most common sleep disorders in PD. Dopaminergic treatments may improve sleep in PD patients; dopamine agonists (DAs), levodopa (LD), and monoaminoxidase B inhibitors (MAOB-I) have been demonstrated to be efficacious in treating sleep disorders in PD. However, ameliorating sleep impairment in PD patients can be challenging for clinicians, and further proof of the need to improve sleep is currently needed. Sleep-related symptoms, among which insomnia and sleep fragmentation are the most frequent sleep complaints in PD patients, are indeed related to a worse quality of life for patients and family members or caregivers [4,5]. A possible mechanism at the basis of the burden of sleep problems in these patients may be related to a degeneration of brainstem centers, such as locus coeruleus and raphe dorsalis, and hypothalamic regions controlling the sleep–wake cycle and ensuring the sleep–wake rhythm by increasing alertness during the day and promoting sleep during the night [6]. Sleep dysregulation is often an early feature of the disease and can also represent a risk factor for the motor progression and the non-motor symptoms (NMS) burden [5,7]. Indeed, RBD and EDS are premotor symptoms, increasing the risk of conversion to PD. Moreover, once the motor symptoms are already evident, poor sleep is associated with increased disease severity and poor quality of life; moreover, nocturnal motor symptoms and fragmented sleep tend to worsen during the progression of the disease [5]. Considering this interconnection between sleep quality and motor symptoms, the concept of “sleep benefit” was described in the past since 33–55% of PD patients can report it [8,9,10]; however, this phenomenon, characterized by the improvement of motor symptoms after a night of sleep, is not entirely understood [8]. Consistently, the “sleep benefit” has not yet been associated with improved sleep structure or continuity. However, it has been hypothesized that a more stable and continuous sleep can permit the improvement of motor symptoms at awakening and increase the response to the anti-Parkinsonian treatment.

Restoring sleep quality and ensuring sleep continuity are two needs of PD patients that should be targeted in clinical practice to improve a patient’s well-being. Other than sleep quality and continuity, head and body positions are essential for permitting the beneficial effects of sleep. Literature data show that PD patients sleep more frequently in the supine position than healthy controls and less often change their head and body position during sleep [11]. This data has been considered with a relevant clinical implication concerning the association of the supine position during sleep with the risk for apnea or hypopnea events and, thus, EDS related to sleep-disordered breathing [12]. Moreover, the recently demonstrated critical role of the glymphatic system (GS), active during sleep for maintaining brain health, increases the importance of monitoring sleep quality and continuity and head and body position during sleep. The GS represents a pathway in charge of cleaning the interstitial waste from the brain parenchyma during sleep [13], and it is controlled by the brain’s arousal level [13]. In keeping with the literature evidence, brain interstitial space volume expands significantly during sleep compared with the awake state. The enlarged brain interstitial space volume during slow wave sleep (SWS) permits the perivascular inflow, increasing the cerebrospinal-fluid (CSF)–interstitial-fluid (ISF) exchange and supporting the transportation of waste solutes to the cerebral veins and then to the cervical lymphatic vessels [13]. This glymphatic transport is more efficient in the lateral than in the supine position [14]. Coupling the literature data about GS functioning with the aforementioned evidence about the “sleep benefit” effect in PD and the more frequent supine position during sleep of PD patients, one could argue about a hypothetical GS malfunction also in PD, although this hypothesis should be tested and validated.

Monitoring sleep with wearable devices in patients’ home environments is a crucial advancement in sleep medicine and clinical research [15]. This approach allows for continuous monitoring over consecutive nights, granting valuable insights into the natural sleep patterns of individuals without the discomfort of a hospital setting. Unlike traditional polysomnography, these devices are far more comfortable and less obtrusive, eliminating the need for bulky systems that can interfere with a patient’s sleep [16]. The ability to assess sleep architecture and head positions in the familiar surroundings of one’s home offers a more accurate reflection of real-life conditions [17]. Considering that several studies have shown the usability of wearable devices in home settings in patients with PD, either in the early or moderate-advanced stages of the disease [17,18], for this study, sleep and head position were monitored by using a novel and validated wearable device (Sleep Profiler headband) [19]. This innovative technology opens new avenues for personalized care and a deeper understanding of the relationship between sleep and PD symptoms.

Therefore, considering the potential role of head and body position during sleep in PD patients and the importance of personalizing the clinical practice by educating patients about head and body position during sleep, also considering the hypothetical link between the “sleep benefit” effect to the GS function, the present exploratory study aimed at evaluating both sleep architecture and head position during each sleep stage in PD patients. Moreover, this study correlated (1) sleep architecture to head position and (2) sleep macrostructure and head position to motor impairment and NMS.

## 2. Methods

### 2.1. Participants and Study Design

This prospective observational study included patients with idiopathic PD admitted at the PD Centre of the University Hospital of Rome “Tor Vergata”. Eligible patients were diagnosed with idiopathic PD according to the criteria released by the Movement Disorder Society [20]. Patients were also required to meet the following inclusion criteria: (1) in case of anti-Parkinsonian treatment, maintaining a stable dose of their medications for at least one month preceding the inclusion in the study; (2) no cognitive impairment, defined by Mini-Mental State Examination (MMSE) score ≥ 24; (3) Hoehn and Yahr (H&Y) stage between 1 and 3. The exclusion criteria were: (1) concomitant neurologic and/or psychiatric diseases evaluated by the anamnestic interview; (2) sleep disorders, such as sleep-disordered breathing, previously diagnosed; (3) use of sleep medications or other treatments influencing sleep (expect anti-Parkinsonian treatments); (4) orthopedics surgery, pain, or conditions not permitting head and body movements during sleep.

All participants underwent sleep recordings by using the Sleep Profiler; in addition, Unified Parkinson’s Disease Rating Scale II and III sections (UPDRS II, UPDRS III) for motor experiences of daily living and motor examination. Clinical evaluation was performed in “ON state”, under the effect of habitual antiparkinsonian therapy. The NMS scale (NMSS) was used for non-motor disturbances; it is a 30-item scale including the following nine domains: cardiovascular, sleep/fatigue, mood/cognition, perceptual problems, attention/memory, gastrointestinal, urinary, sexual function, and miscellaneous (pain, smell, weight, and sweating) [12]. NMSS was performed at awakenings the last day of recording.

The following data were then collected: sex, age, disease duration, disease phenotype (akinetic-rigid, tremorigen, and mixed), body mass index (BMI), and anti-Parkinsonian treatment, counted as Levodopa Equivalent Daily Dose (LEDD, mg/day), which was calculated in each patient using conventional formula.

The study was approved by the local Ethics Committee and was conducted according to the Helsinki Declaration of 1975. All the participants provided their signed informed consent.

### 2.2. Sleep Profiler

All patients were instructed to wear the Sleep Profiler device at home for up to three consecutive nights. This device, validated as an adequate alternative to full polysomnography [19,21], permits the recording of electroencephalography (EEG), electrooculography (EOG), and electromyography (EMG) from three frontopolar EEG signals. It is also composed of an accelerometer to detect head positions, a microphone for the quantitative estimation of snoring, and an optical sensor to assess pulse rate.

The data acquired were analyzed by an autoscoring software that decomposes the EEG signals into power spectral bands and then allows recognition of the sleep stages in 30 s epochs. Moreover, the Sleep Profiler automatically calculates sleep onset latency (SOL), total sleep time (TST), sleep time in the supine position, wake after sleep onset (WASO), and sleep efficiency (SE) from EEG channels as well as sleep time spent in each of the four sleep stages: stage 1 of Non-REM sleep (N1), stage 2 of Non-REM sleep (N2), stage 3 of Non-REM sleep (N3), and REM. Finally, it also recognizes SWS and slow wave activity (SWA). All these stages were also calculated considering the head position. Data were checked by experienced researchers in sleep medicine (CL, CC, MF) to control the automatic analysis performed by the software.

### 2.3. Data Analysis

In addition to the sleep parameters, we extracted the total recording time in the supine, lateral left, lateral right, prone, and upright positions. The percentage of SWA, N3, and REM in the supine position was also calculated and included in the correlation results. First, descriptive statistics were performed to characterize the dataset. Then, we calculated the statistical differences across the nights, the distribution over the nights of the different sleep positions, and the sleep stages in the supine position. The non-parametric statistical test, the Mann–Whitney U test, was used for the not normally distributed data. We also evaluated Spearman’s correlation coefficient between the clinical data and the sleep parameters.

Data extraction, analysis, and correlation were performed using Python 3.9.13 (Python Software Foundation), and the *p*-value was set at *p* < 0.05 for statistical significance.

## 3. Results

### 3.1. Demographical and Clinical Data

The study included twenty patients diagnosed with PD, of which 50% were male, with a mean age of 65.7 ± 8.6 years. At the outset, PD patients exhibited a mean UPDRS-III score of 24.1 ± 9.1, a mean H&Y score of 1.85 ± 0.6, and a mean MMSE score of 28.2 ± 1.7. Phenotypically, 30% of the patients displayed tremor-dominant symptoms, 40% exhibited akinetic-rigid symptoms, and 30% presented a mixed phenotype. The participants’ demographic and clinical characteristics at baseline are presented in Table 1.

### 3.2. Sleep Data

**Figure 1:**
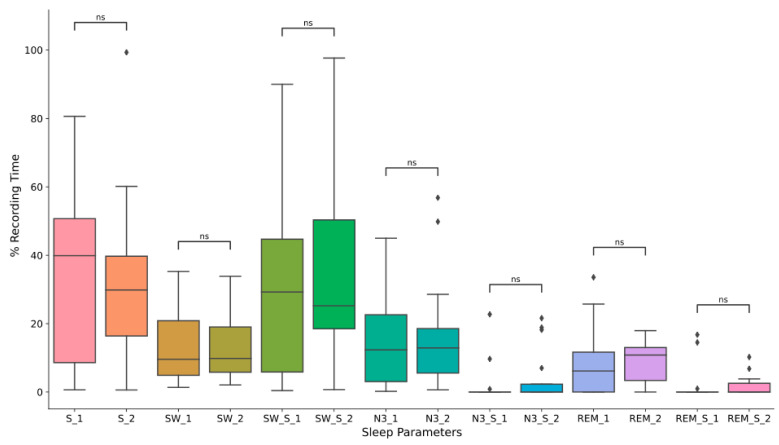
Graphical representation of sleep parameters in the last two nights of recording (*n* = 17). _1 = second last night, _2 = last night. S = supine, SW = slow waves, SW_S = slow waves in the supine position, N3 = stage 3 of non-REM sleep, N3_S = stage 3 of non-REM sleep in the supine position, REM = REM sleep, REM_S = REM sleep in the supine position, ns = not significant. The diamonds represent the outliers.

Eleven patients wore the Sleep Profiler device for three consecutive nights, six for two consecutive nights, and three for one night. Comparative analysis of sleep data between two and three consecutive nights did not reveal significant differences in sleeping posture or sleep architecture parameters, as depicted in Figure 1. Further statistical details are available in Supplementary Table S1. Consequently, for the analysis of sleep parameters, we considered the average of the last two nights for the seventeen participants with multiple consecutive nights, including the three patients who recorded data for only one night. The sleep data collected from all twenty patients were subsequently correlated with their clinical features.

**Figure 2:**
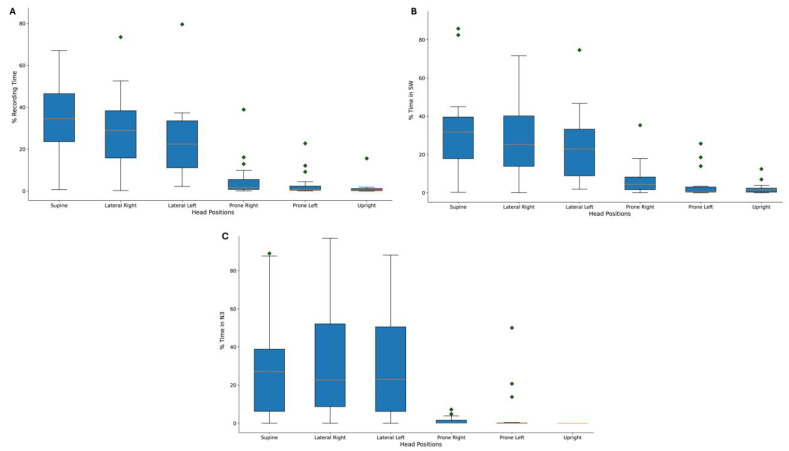
(**A**) Distribution of the head positions during the sleep recording. (**B**) Slow wave distribution over the different head positions during the sleep recording. (**C**) Stage 3 of non-REM sleep distribution over the different head positions during the sleep recording. The diamonds represent the outliers.

We observed a trend for the PD population to sleep more frequently in the supine head position compared to other head positions, as illustrated in Figure 2A. The sleep architecture of the PD patients in this study is comprehensively presented in Table 1.

Notably, it was evident that SWA and N3 sleep stages were more predominant in the supine head position compared to other positions, as demonstrated in Figure 2B,C.

**Figure 3:**
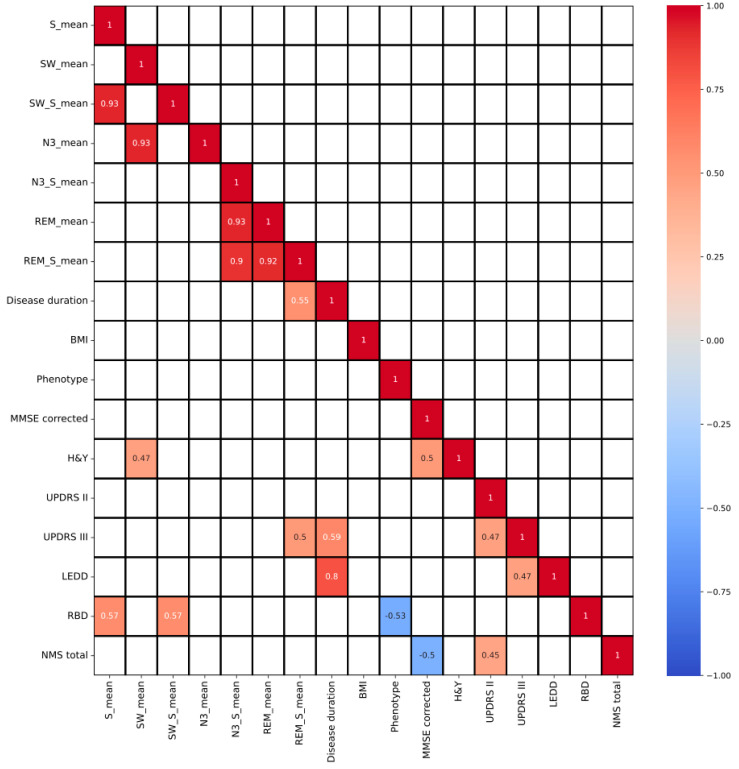
Heatmap, correlation matrix. Only values with a *p*-value < 0.05 were highlighted. S_mean = average time spent in supine position; SW_mean = average time in slow wave; SW_S_mean = average time in slow wave and supine position; N3_mean = average time in N3; N3_S_mean = average time in N3 and supine position; REM_S_mean = average time in REM and supine position.

Spearman’s correlation analysis, shown in Figure 3 and represented as a heat map, revealed associations between clinical measures and sleep parameters. Notably, N3 sleep in the supine position strongly correlated with REM sleep in the same position (correlation coefficient = 0.90, *p*-value < 0.01). Furthermore, REM sleep in the supine head position correlated with disease duration (correlation coefficient = 0.48, *p*-value = 0.03) and UPDRS III scores (correlation coefficient = 0.53, *p*-value = 0.02).

The presence or absence of anti-Parkinsonian medications, specifically LEDD, was significantly correlated with UPDRS-III scores (correlation coefficient = 0.81, *p*-value = 0.00002) and disease duration (correlation coefficient = 0.50, *p*-value = 0.03). However, no significant correlation was found between LEDD and sleep position.

Twelve out of the twenty PD patients in this study were diagnosed with RBD. The presence of RBD significantly correlated with the time spent in the supine position (S_mean) (correlation coefficient = 0.57; *p* = 0.009) and the time spent in SWS during the supine position (SW_S_mean) (correlation coefficient = 0.57; *p* = 0.009).

To further investigate the data, we divided the dataset of 20 PD patients into two groups based on the amount of N3 sleep spent in the supine position. A median value was calculated to separate patients into groups with N3 above or below the median. This division revealed significant differences between the two groups regarding disease duration (*p*-value 0.009) and UPDRS-III scores (*p*-value 0.034). Similar results were obtained when grouping patients based on the time spent in REM sleep and the supine position.

## 4. Discussion

This pilot study was set to investigate the sleep characteristics of patients with PD, focusing on the head and body positions during each sleep stage and the importance of maintaining the activity and function of the GS in patients with neurodegenerative disorders [8,10,11,12]. Therefore, considering the high prevalence of sleep disorders in patients with PD, adding further information about sleep is essential for clinical practice. Sleep disturbances are indeed frequently encountered NMS in PD patients. They may be present in the premotor stages of the disease or along the disease progression. Recent evidence suggests that more than 90% of PD patients complain for primary or secondary sleep disorders. In particular, anti-Parkinsonian treatment has been associated with the appearance of secondary sleep disorders; accordingly, EDS has been frequently related to antidopaminergic treatment. The present study confirmed that PD patients sleep prevalently in the supine position, and SWA and N3 were more represented in the supine position. The here reported findings further show that PD patients present the unchanging sleep architecture across consecutive nights. Moreover, patients with PD maintain stable head positions during sleep since the time spent in the supine position remains stable across the nights. These results are relevant for clinicians who should ask about the sleep habits of PD patients, considering that head and body position can interfere with the beneficial effects of sleep. Indeed, recent reviews showed experimental and epidemiological data trying to determine the relationship between GS dysfunction, sleep disturbance, and PD pathogenesis and progression [22]. GS is recognized as a pathway essential for maintaining brain health during sleep, and it is supposed to fail in functioning in patients with neurodegenerative disorders [13].

At the sleep architecture analysis, as expected, N2 was the more frequent sleep stage [23]. Considering the time spent in the different positions during sleep, SWA and N3 stages were performed more frequently in the supine position. Moreover, the time spent in the supine position during REM and N3 strongly correlated, further documenting the continuity of sleep performed in the supine position with a reduced rate of body position changes, already described in PD patients [12,24]. Persons usually change their sleeping position 11 times per night, but there was no evidence about the number of position changes during sleep in neurodegenerative disorders.

Considering the head position of PD patients during sleep, the analysis documented that N3 and SWA were more frequently performed in the supine position. This is the most important result of this study since it can add to the supposed high risk of GS malfunction in patients with PD, considering the results of the present study showing the following: (i) PD patients tend to sleep more frequently in the supine head position; (ii) patients more frequently performing N3 sleep in the supine head position presented higher UPDRS-III (motor) scores than those sleeping in the N3 less frequently in the supine head position; (iii) disease duration correlated to the more frequent REM sleep in the supine position. Taking all these results into account and considering that the GS is more active during the SWS and N3 stages but is less functional in the supine position, it is only supposed the GS dysfunction in PD patients at the basis of the association between supine head position and motor impairment. Consistently, the glymphatic transport is more efficient in the lateral position than in the supine or prone one [14]. The mechanisms at the basis of this reduced functionality and the clinical implications of this finding are not entirely understood, and literature uniquely proposes some hypotheses drawn from animal model studies and not confirmed in human studies [14]. The principal suggested mechanism behind the effects of head and body position on brain clearance is the reduced venous drainage due to gravity, which predisposes the collapse of the vessels in the supine position. An unproven hypothesis is related to the influence of sleep position on the breathing patterns in the sleeping state [25,26] since a lower respiratory effort could reduce vascular pulsatility, resistance, and the glymphatic flow [27]. A final hypothesis is related to the autonomic unbalance between the orto- and para-sympathetic networks, favoring the orto-sympathetic system in the supine position and the para-sympathetic system in the lateral decubitus. When the orto-sympathetic network overcomes in the supine position, the norepinephrine levels increase and thus reduce the GS function since norepinephrine is a potent inhibitor of the glymphatic influx [28].

Hence, it was suggested that lateral decubitus during sleep, particularly during the N3 stage, is essential for the correct functioning of the GS. This supposed evidence is in contrast with the findings that PD patients sleep more frequently in the supine position, and their N3 stage is more commonly performed in the supine position. Therefore, the possibility of GS malfunction in PD patients can be suggested, although should be tested in future studies, and can further increase the risk for the progression of the neurodegenerative process due to the loss of functioning of the GS.

The total time spent in the supine position is considered a risk factor for GS functioning instead of the number of position changes during the night [29]. Considering that N3 and SWA were more frequently performed in the supine position, this result further substantiates the supposed high risk of GS malfunctioning in patients with PD, considering that animal studies documented that the GS is more active during the SWS and N3 stages, but it is impaired by the supine position.

Considering the correlations between motor impairment, disease severity, and the sleep stages, also concerning the head position, the time spent in REM in the supine position correlated positively with the longer disease duration and the motor disability measured by the motor section of the UPDRS. This result confirmed the immobility during sleep in the supine position occurring in PD patients, which is more evident in the moderate-advanced stages of the disease [11].

Hence, the novel, although preliminary, results of this study showed that the crucial sleep stages (N3 and SWA) for GS functioning are performed in the supine head position by PD patients, who tend to maintain a stable sleep architecture across the nights without significantly changing the head position during sleep. These findings highlight the importance of monitoring sleep in PD patients in a personalized approach based on asking about total sleep time and considering head and body positions. Consistently, in the recent past, it appeared evident that sleep, specifically deep sleep (SWA, N3, and REM stages), enhancement can represent a therapeutic strategy against the neurodegenerative processes; accordingly, sleep improvement should be included in the setting of disease-modifying therapeutic strategies.

Limitations of the present study are the small group of patients, substantiating the exploratory nature of the study, and the need to confirm these preliminary results in future investigations. Moreover, sleep was exclusively monitored by the Sleep Profiler headband, validated in previous studies [19], but without giving the complete picture of sleep architecture and breathing during sleep, thus increasing the risk of underestimating sleep-disordered breathing. It is known that in PD subjective sleep complaints and objective PSG measurements often show discrepant results. Furthermore, questionnaires were designed to record sleep disturbances over a longer time periods while polysomnographic or actigraphic parameters reflect only single or few measurements. Notably, subjective scales reflect the chronic effect of treatment whereas polysomnography or actigraphy may show exclusively the single- or few-night sleep status. Moreover, the absence of a control group can further limit the significance of the present results, that should be confirmed in further studies.

In this study, the use of a wearable device, the Sleep Profiler, marked a significant advancement in sleep research in PD patients. This technology allowed us to non-intrusively monitor patients’ sleep patterns and head positions in their homes up to three consecutive nights. This approach provided several distinct advantages. Firstly, it enabled us to investigate sleep architecture and head position in a familiar and less clinical environment, reducing the likelihood of altering the natural sleep of our study participants. This home-based approach is precious in studying chronic conditions like PD, where sleep disturbances are part of daily life. Secondly, by monitoring patients across multiple nights, we gained insights into the consistency and stability of sleep patterns within this population. Lastly, the Sleep Profiler effectively bridged the gap between traditional laboratory polysomnography and actigraphy, offering a comprehensive assessment of sleep architecture and head position dynamics in PD patients, opening up new avenues for future research and personalized care strategies.

In conclusion, this pilot study confirmed the previous evidence that PD patients sleep more frequently in the supine position and tend to maintain stable sleep characteristics and body and head positions during sleep across the nights of recording. The novel, although preliminary, results are the evidence that the crucial sleep stages (N3 and SWA) for GS functioning are performed in the supine position by PD patients; consequently, the hypothesis of GS dysregulation in PD due to the sleep (mainly N3 and SWA) performed in the supine head position should be evaluated also for possibly explaining the “sleep benefit” effect, previously described and not completely explained by the literature. These results highlight the importance of monitoring sleep in PD patients, asking about total sleep time, and considering head and body positions. Consistently, in the recent past, it appeared evident that sleep promotion, specifically deep sleep (SWA, N3, and REM stages), can represent a target for disease-modifying therapeutic strategies. Therefore, this study proposes not only to sleep enhancement as a therapeutic target but also sleep position monitoring to ensure sleep’s beneficial effects in PD better. Notably, considering the importance of monitoring NMS and then sleep in PD patients [30], the body and head positions should be checked during sleep since maintaining the lateral decubitus is crucial to promote the beneficial effects of sleep mediated by the GS activity.

## Figures and Tables

**Table 1:** Demographic, clinical, and sleep data of PD patients.

	PD Patients (*n* = 20) (Mean ± SD)
**Demographic Data**	
Mean Age (y.o.)	65.7 ± 8.6
Female	10 (50%)
BMI	26.59 ± 3.5
**Phenotype**	
Tremorigen	6 (30%)
Akinetic-rigid	8 (40%)
Mixed	6 (30%)
Disease duration (years)	3.9 ± 3.5
UPDRS-III mean score	24.1 ± 9.1
UPDRS-II mean score	10.55 ± 5.7
H&Y mean score	1.85 ± 0.6
NMSS total score	54.45 ± 35.7
LEDD	271.2 ± 383
**Cognitive Data**	
MMSE	28.2 ± 1.7
**Sleep Architecture**	
TST [Hours]	5.1 ± 1.7
SE [%]	69.9 ± 18.4
SOL [Minutes]	22.8 ± 13.3
Wake [%]	30.2 ± 13.8
REM [%]	8.4 ± 6.2
N1 [%]	5.8 ± 11.8
N2 [%]	24.9 ± 11.8
N3 [%]	18.4 ± 15.2
Invalid [%]	1.1 ± 1.6

PD = Parkinson’s disease; BMI = Body Mass Index; UPDRS II-III = Unified Parkinson’s Disease Rating Scale II and III sections; H&Y = Hoen and Yahr NMSS = Non-Motor Symptoms Scale; LEDD = Levodopa Equivalent Daily Dose; MMSE = Mini-Mental State Examination; SD = Standard Deviation; TST = Total Sleep Time; SE = Sleep Efficiency; SOL = Sleep Onset Latency.

## Data Availability

The data that support the results reported in this study are available from the corresponding author upon reasonable request.

## Supplementary Materials

The following supporting information can be downloaded at: https://www.mdpi.com/article/10.3390/jpm13111591/s1, Table S1: Descriptive data of Figure 1.
