# Supplementary material for: Exploring the Association Linking Head Position and Sleep Architecture to Motor Impairment in Parkinson’s Disease: An Exploratory Study
Source: J Pers Med. 2023 Nov 10;13(11):1591. doi: 10.3390/jpm13111591 (PMC10671918; doi:10.3390/jpm13111591)
Supplement: Supplementary file 1 [file jpm-13-01591-s001.zip › jpm-2593349-supplementary.pdf]

Supplementary Table S1. Descriptive data of Figure 1.

.	Night 1: mean ± SD	Night 2: mean ± SD	p-value
%_S	36.7 ± 26.2	32.5 ± 24.1	0.51
%_SW	12.8 ± 10.1	13.3 ± 9.7	0.70
%_SW_S	33.2 ± 27.3	35.3 ± 26.7	0.86
%_N3	14.3 ± 13.4	16.5 ± 15.9	0.71
%_N3_S	1.9 ± 5.8	4.1 ± 7.6	0.18
%_REM	8.6 ± 10.1	9.1 ± 6.3	0.36
%_REM_S	1.9 ± 5.2	1.7 ± 2.9	0.21

% of total recording time spent in supine position (%_S); in slow wave (%_SW); in slow wave and supine position (%_SW_S); in non-REM 3 (%_N3); in non-REM 3 and supine position (%_N3_S); in REM (%_REM); in REM and supine position (%_REM_S); for the 17 PD patients wearing the Sleep Profiler two or three consecutive nights.
